# Multimodal brain data and core dimensions of creativity

**DOI:** 10.1016/j.dib.2020.105176

**Published:** 2020-02-04

**Authors:** Jordan Poppenk

**Affiliations:** a Department of Psychology, Queen's University, Kingston, ON, 62 Arch St., K7L 3N6, Canada; b Centre for Neuroscience Studies, Queen's University, Kingston, ON, 18 Stuart St., K7L 3N6, Canada; c School of Computing, Queen's University, Kingston, ON, 557 Goodwin Hall, K7L 2N8, Canada

**Keywords:** Creativity, Resting-state fMRI, Structural MRI, Diffusion imaging, Individual differences

## Abstract

The current dataset incorporates multimodal brain imaging and creativity test data from a sample of 66 healthy young adults, all of whom were healthy right-handed English speakers, aged 22 to 35, with normal or corrected-to-normal hearing and vision. The participants completed measures of divergent thinking (Abbreviated Torrance Test for Adults; ATTA), everyday creativity (Creative Behaviour Inventory; CBI), and creative achievement (Creative Achievement Questionnaire; CAQ), consistent with the known multidimensional nature of creativity. They also completed high-resolution anatomical scans (T1-weighted and T2-weighted), diffusion tensor imaging scans, and resting state fMRI scans. The data were originally used in the article *Neuroimaging predictors of creativity in healthy adults* by Sunavsky and Poppenk [1] to test a set of confirmatory predictions regarding the volumetric, structural connectivity, and functional connectivity correlates of creativity. The data are uniquely high-dimensional in measuring both multiple dimensions of creativity as well as multimodal brain data, and may be valuable to researchers for testing models of individual differences in creativity, or who are seeking to integrate multiple datasets for large-scale, multi-site analysis of creativity.

**Table:** Specifications Table

Subject	Cognitive Neuroscience
Specific subject area	Creativity
Type of data	Table Brain images
How data were acquired	Siemens whole-body 3T fMRI scanner Abbreviated Torrance Test for Adults (ATTA); Creative Behaviour Inventory (CBI); and Creative Achievement Questionnaire (CAQ)
Data format	Raw
Parameters for data collection	Data collection incorporated the following parameters. 1) Participant demographics (age 22 to 35): right-handed, normal or corrected-to-normal vision and hearing, no history of psychological or neurological disorders, no substance abuse, able to keep still in a mock MRI scanner with no claustrophobia, able to perform above chance on memory tests, and voluntary participation; 2) neuroimaging protocol (Appendix A); 3) Behavioural testing environment (computer in a laboratory-controlled setting).
Description of data collection	In a controlled laboratory setting with a computer, participants completed the ATTA, CAQ and CBI. In a separate session, participants returned for MRI scanning. The day prior to MRI scanning, participants completed 45 minutes of motion biofeedback training, both to become better habituated to an MRI-like environment, and to learn to reduce their head movement.
Data source location	Queen's University Kingston, Ontario Canada
Data accessibility	Repository name: openneuro.org Data identification number: 10.18112/openneuro.ds002330 Direct URL to data: https://openneuro.org/datasets/ds002330
Related research article	Sunavsky, A., & Poppenk, J. (2019). Neuroimaging predictors of creativity in healthy adults. *NeuroImage*. https://doi.org/10.1016/j.neuroimage.2019.116292

**Table:** 

**Value of the Data** • The current dataset provides a link between different known dimensions of a person's creativity on the one hand, and the anatomical and functional structure of that person's brain on the other. • Cognitive neuroscience researchers may find the data valuable for elucidation of the neural substrates of creativity. • This dataset can be used by researchers developing more sophisticated neural models of creativity who are in need of data for testing those models. • This dataset is uniquely high-dimensional in measuring both multiple dimensions of creativity, their facets, and multimodal brain data. • The current sample can be combined with others to establish the larger sample sizes needed for testing more complex neurocognitive models.

## 1 Data description

The current dataset was first used in an analysis on the neural substates of creativity [1], but invites a range of possible further uses. It is hosted on openneuro.org [2] and is organized using BIDS format [3], which specifies the naming conventions and folder organization for cognitive and neuroimaging files. The nature of the files included is described below.

### 1.1 Cognitive data

A tab-separated file (participants.tsv) describes participants' demographic and creativity characteristics. Each participant is listed by a code number (sub-01 through sub-66). Demographic data fields include age and sex (male, female, or other). Cognitive scores are reported in this same table, with an additional column for each summary creativity measure. Building on the notion that creativity is multifaceted, measures of everyday creative behaviour, creative achievement, and divergent thinking were sampled and included. These were measured using the Creative Behaviour Inventory (CBI) [4], the Creative Achievement Questionnaire (CAQ) [5], and the Abbreviated Torrance Test for Adults (ATTA) [6]. The CBI score corresponds to a self-report estimate of the participant's tendency to habitually engage in a variety of creative behaviors in everyday life. The nature of the data from the other two tests is more complex and is described below.

As a measure of participants' accomplishments, the CAQ involves self-report questions that address whether the participant has attained a particular creative achievement. These questions are posed in a series of domains (e.g., music, comedy, theatre), and evaluated to generate a domain achievement score. The domains are also combined to create an overall achievement score (see *Experimental Design, Materials, and Methods* for scoring details). Both domain-specific achievement, as well as overall achievement, are reported in the participants.tsv data file. The specific domains assessed and included were: architectural design, creative writing, culinary arts, humor, inventions, music, scientific discovery, theater and film, and visual arts. As individual items within these domains potentially could reveal identifying information (e.g., distinctive accomplishments), the data are presented as summary scores at the domain level.

As a measure of divergent thinking, the ATTA is provided. The ATTA has been validated as an abbreviated version of the Torrance Test for Adults [7], which is a widely used test of divergent thinking. Typically, separate scores are evaluated in both verbal and visual domains, with a composite creativity score computed from rater evaluations of various facets. As with the CAQ, data have been included in the table for individual dimensions (see *Experimental Design, Materials, and Methods* for scoring details).

For each of verbal and visual creativity, different criterion-referenced measures were assessed to obtain composite scores, following the detailed scoring guidelines described in the ATTA scoring guide [6]. For the verbal ATTA, these dimensions evaluated the presence of: conceptual incongruity (considered an objective basis for the presence of humor), emotions and feelings, future orientation, provocative questions, and richness and colorfulness of imagery. For the visual ATTA, these included the presence of: an abstract design, an abstract title, emotion, fantasy, an internal visual perspective (e.g., revealing the inner workings of something), movement and/or sound, openness (resistance to visually closing objects), richness and colorfulness of imagery, synthesis, unusual perspective (other than head-on), and visual context. Further to these criterion-referenced measures, norm-referenced measures were also computed for ATTA: for verbal ATTA, these included originality and fluency. For the visual ATTA, these included originality, fluency, elaboration, and flexibility.

## 2 Neuroimaging data

Several types of neural data are included in the dataset: for each participant, there are T1-weighted and T2-weighted anatomical images, diffusion-weighted images, functional neuroimaging data, and single-band reference images needed for dewarping of the neuroimaging data (dataset). Data are organized into participant folders named according to the same coding system described in the cognitive data table. For each type of neuroimaging data, .json files containing metadata may be found at the root level of the dataset hierarchy. These metadata files include technical details needed for most analysis as a convenience. Supplemental to these files, a more comprehensive set of technical parameters may be found in the appendix [8].

Within each participant folder, four subfolders may be found. In the anat folder are located the detailed T1-weighted and T2-weighted whole-brain neuroanatomical scans (sub-##_T1.nii.gz and sub-##_T2.nii.gz, respectively). Each type of anatomical scan is sampled at a 0.7 mm^3^ isotropic resolution, with facial features manually removed to maintain participants' anonymity. At the root level of the dataset are two files that describe important scanner parameters pertinent to analysis of all participants' anatomical data: T1w.json and T2w.json, describing parameters for the T1-weighted and T2-weighted scans, respectively.

In the dwi folder, 64-direction diffusion-weighted whole-brain images are stored together with three b0 scans. Each diffusion imaging series features 1.5 mm^3^ isotropic resolution. One series was collected with a left-to-right acquisition direction (sub-##_acq-lr_dwi.nii.gz), and one with a right-to-left acquisition direction (sub-##_acq-lr_dwi.nii.gz). Each of these series was accompanied by both a bvecs file (sub-##_acq-lr_dwi.bvec) and a bvals file (sub-##_acq-lr_dwi.bval). At the root level of the dataset are two files that describe important scanner parameters pertinent to analysis of all participants' diffusion imaging data: acq-lr_dwi.json and acq-rl_dwi.json, describing parameters for the left-to-right and right-to-left acquisition series, respectively.

The fmap and func folders both contain neuroimaging files related to the resting-state connectivity scans. The func folder of each participant contains two multiband fMRI runs, each consisting of 158 functional volumes acquired over a 1.9 s TR, for a total run duration of 5 min each. The images feature 1.5 mm^3^ isotropic resolution. Scanning parameters for the two runs are found in the task-rest_bold.json file found at the root level of the dataset. By contrast, the fmap folder contains single-band reference scans for both anterior-to-posterior and posterior-to-anterior fMRI acquisitions (sub-01_dir-ap_epi.nii.gz and sub-01_dir-ap_epi.nii.gz, respectively). As with the dwi folder, at the root level of the dataset are two files that describe scanner parameters pertinent to analysis of all participants' functional imaging data: dir-ap_epi.json and dir-pa_epi.json, describing parameters for the anterior-to-posterior and posterior-to-anterior acquisition series, respectively. These scans and parameters can be used to generate a dewarping map for the main functional timeseries.

## 3 Experimental Design, materials, and methods

### 3.1 Participants

104 participants were recruited using posters, email, and social media. Participants were required to be aged 22–35 years, after [9]. They were also required to be right-handed, have normal or corrected-to-normal hearing and vision, have no history of substance abuse or psychological or neurological disorders, and to have no contraindications for MRI scanning. Participants claiming by email or phone to meet these characteristics were invited for further screening. In an initial section, it was confirmed that participants had acceptable reading ability by administering the TOWRE Nelson-Denny score (screening for minimum scores of scores of 26.4 and 2, respectively), ability to follow instructions (as assessed by scoring above chance on a cognitive and memory task), and ability to stay still for 20 minutes without falling asleep and without claustrophobia inside a simulated neuroimaging scanner. Participants were allowed to continue if they did not express rudeness or have multiple no-shows. After screening, a total of 69 participants were retained for further experimentation, of whom 66 completed all tasks. All procedures were approved by the Queen's Health Research Ethics Board.

### 3.2 Cognitive testing

The current data were gathered as part of a larger protocol in which participants returned for multiple visits. Over two separate sessions, the CBI [4]; the CAQ [5]; and the ATTA [6] were all administered. This behavioural testing was completed using Linux desktop computers (Ubuntu 2014.04 LTS) featuring a 23.8” IPS monitor (resolution: 1920 × 1080 pixels) in a computer testing room. The experiment was run using MATLAB (2016a, The Mathworks) for Linux with Psychtoolbox [11] and SuperPsychToolbox [12].

Administration of CBI [10] consisted of participants completing a questionnaire on a computer about their creative behaviors. The 77-item version of this scale was used. Each item describes engagement in a unique creative behavior (e.g., “Designed and made your own greeting card.”). For each item, participants indicated how many times they had engaged in that behaviour. A score was later assigned to each item based on their self-reported frequency of engaging in that behaviour: “Never did this” (zero points); “Did this once or twice” (one point); “3–5 times” (two points), or “More than 5 times” (three points). The mean was taken across scores for all items to obtain a single overall score for each participant.

Administration of the CAQ involved participant self-report regarding whether different achievements had been attained in ten creative domains (listed under *Data description*; see also appendix of [5], section 2 for individual items and scoring). Questions progressed through each domain. The first item within each domain addressed a low-level achievement. If the participant indicated they had met this low level of achievement, questions advanced to higher levels of achievement for that same domain. Otherwise, questioning moved onto the next domain. Standard scoring was applied [5], with more points being assigned for more substantial accomplishments, and more points being assigned for certain accomplishments that had been attained multiple times. For each participant, the sum of points within each domain was computed to obtain a domain score, and was then taken across all domains to obtain a single creative achievement score.

Turning to the ATTA (2002 version), administration began with participants writing a response to an unusual situation in which they described possible problems that could arise in that situation. Next, participants were presented with a shape, and asked to complete a drawing that incorporated it. Finally, they were presented with an array of shapes, and again asked to complete a drawing involving them. Two raters scored each of these responses following the standard procedures described in the ATTA scoring guide [6]. In particular, fifteen criterion-referenced measures (five verbal, ten visual, as listed above under *Data description*) were assigned a score of zero, one (“single-plus”), or two (“double-plus”) based on raters' impressions of the degree to which the relevant feature was present in the participants' written or drawn response. In addition, two verbal and four visual norm-referenced measures were collected in which tallies of features were scaled based on typical rates in the population. For a *fluency* measure, raters counted the number of ideas present in the written response or drawing; for an *originality* measure, raters counted the number of atypical ideas in the written response or drawing; for an *elaboration* measure, raters counted the number of embellishments made to a drawing; and for a *flexibility* measure in the final array drawing, raters counted the number of times instances among the array of shapes were used in different ways in a drawing.

The above criterion- and norm-referenced sub-scores were used to generate two main ATTA summary measures of interest: total verbal creativity and total visual creativity. A sum was taken of all scores among the scaled verbal norm-referenced measures and verbal criterion measures to generate a verbal creativity score, and a similar addition was performed among visual measures to compute a total visual creativity score. Agreement across raters was high, with an Intraclass Correlation Coefficient (ICC) of 0.903 for verbal creativity scores, and 0.943 for visual creativity scores. The average score was therefore taken across raters for each participant to obtain a verbal creativity and visual creativity score.

### 3.3 Neuroimaging

Neuroimaging took place after behavioral testing was complete. The day before their neuroimaging appointment, participants completed a motion biofeedback session in which they were shown a 45-minute nature documentary inside a mock fMRI scanner. Overlaid on the movie was a live-updating chart depicting a recent (10 second) history of their head motion, as measured using a sensitive inclinometer (SCA121T-D05 Dual Axis). When participants' movements exceeded an adaptive threshold, the film was paused and loud static was played for several seconds. Participants were quizzed about the film afterwards to confirm their engagement in the task.

The following day, participants completed a 1.5 hour scan in a Siemens whole-body 3T fMRI scanner (Magnetom Tim Trio; Siemens Healthcare). The scanning session involved acquisition of structural images, diffusion images and blood-oxygen level dependent (BOLD) images (for protocols, see Ref. [8]). High-resolution whole-brain T1-weighted (T1w) and T2-weighted (T2w) anatomical images were gathered (in-plane resolution 0.7 × 0.7 mm^2^; 320 × 320 matrix; slice thickness 0.7 mm; 256 AC-PC transverse slices; anterior-to-posterior encoding; 2x acceleration factor; T1w: TR 2400 ms; TE 2.13 ms; flip angle 8°; echo spacing 6.5 ms; T2w: TR 3200 ms; TE 567 ms; variable flip angle; echo spacing 3.74 ms). Then, complementary diffusion tensor imaging (DTI) scans were gathered using right-to-left and left-to-right encoding directions (64 diffusion directions; b-value 1200; in-plane resolution 1.5 × 1.5 mm^2^, 128 × 128 matrix; slice thickness 1.5 mm; 93 AC-PC transverse slices; 3 x multiband acceleration factor; TR 5180 ms; TE 103.4 ms; flip angle 78°; echo spacing 0.77 ms). Three b0 scans were gathered for each encoding direction. Resting-state functional images included two T2*-weighted scans (158 vol; in-plane resolution 1.5 × 1.5 mm^2^; 128 × 128 matrix; slice thickness 1.5 mm; 90 AC-PC transverse slices; anterior-to-posterior encoding; 6 x multiband acceleration factor; TR, 1900 ms; TE 36.8 ms; flip angle 75°; echo spacing 0.88 ms).
