# Supplementary material for: Multimodal brain data and core dimensions of creativity
Source: Data Brief. 2020 Feb 4;29:105176. doi: 10.1016/j.dib.2020.105176 (PMC7036717; doi:10.1016/j.dib.2020.105176)
Supplement: Multimedia component 1 [file mmc1.pdf]

\\USER\Dr Poppenk\fmri\MEMRI\BOLD_AP_SB_SE_1.5iso-hippar

TA: 1:02 PAT: Off Voxel size: 1.5×1.5×1.5 mm Rel. SNR: 1.00 USER: cmrr_mbep2d_se

Properties

Prio Recon	Off
Before measurement	
After measurement	
Load to viewer	On
Inline movie	Off
Auto store images	On
Load to stamp segments	Off
Load images to graphic segments	Off
Auto open inline display	Off
Start measurement without further preparation	On
Wait for user to start	Off
Start measurements	single

Routine

Slice group 1	
Slices	90
Dist. factor	0 %
Position	Isocenter
Orientation	Transversal
Phase enc. dir.	A >> P
Rotation	0.00 deg
Phase oversampling	0 %
FoV read	192 mm
FoV phase	100.0 %
Slice thickness	1.50 mm
TR	15530 ms
TE	97.6 ms
Multi-band accel. factor	1
Filter	Raw filter
Coil elements	HEA;HEP

Contrast

MTC	Off
Magn. preparation	None
Flip angle	90 deg
Refocus flip angle	180 deg
Fat suppr.	Fat sat.
Grad. rev. fat suppr.	Enabled
Averaging mode	Long term
Reconstruction	Magnitude
Measurements	3
Delay in TR	0 ms
Multiple series	Off

Resolution

Base resolution	128
Phase resolution	100 %
Phase partial Fourier	7/8
Interpolation	Off
PAT mode	None
Matrix Coil Mode	Auto (CP)
Distortion Corr.	Off
Prescan Normalize	Off
Raw filter	On
Intensity	Weak
Slope	25
Elliptical filter	Off
Hamming	Off

Geometry

Multi-slice mode	Interleaved
Series	Interleaved
Special sat.	None

System

Body	Off
HEP	On
HEA	On
Positioning mode	REF
Table position	H
Table position	0 mm
MSMA	S - C - T
Sagittal	R >> L
Coronal	A >> P
Transversal	F >> H
Coil Combine Mode	Sum of Squares
AutoAlign	---
Auto Coil Select	Default
Shim mode	Standard
Adjust with body coil	Off
Confirm freq. adjustment	Off
Assume Silicone	Off
? Ref. amplitude 1H	0.000 V
Adjustment Tolerance	Auto
Adjust volume	
Position	Isocenter
Orientation	Transversal
Rotation	0.00 deg
R >> L	192 mm
A >> P	192 mm
F >> H	135 mm

Physio

1st Signal/Mode	None

BOLD

GLM Statistics	Off
Dynamic t-maps	Off
Starting ignore meas	0
Ignore after transition	0
Model transition states	On
Temp. highpass filter	On
Threshold	4.00
Paradigm size	15
Meas[1]	Baseline
Meas[2]	Baseline
Meas[3]	Baseline
Meas[4]	Baseline
Meas[5]	Baseline
Meas[6]	Baseline
Meas[7]	Baseline
Meas[8]	Baseline
Meas[9]	Baseline
Meas[10]	Baseline
Meas[11]	Active
Meas[12]	Active
Meas[13]	Active
Meas[14]	Active
Meas[15]	Active
Motion correction	Off
Spatial filter	Off

Sequence

Introduction	Off
Contrasts	1

Bandwidth	1776 Hz/Px
Free echo spacing	Off
Echo spacing	0.88 ms
EPI factor	128
RF pulse type	Normal
Gradient mode	Fast
Fake MB factor for SB	6
SENSE1 coil combine	Off
Invert RO/PE polarity	Off
FFT scale factor	1.00
Physio recording	Legacy
Triggering scheme	Standard

\\USER\Dr Poppenk\fmri\MEMRI\BOLD_PA_SB_SE_1.5iso-hippar

TA: 1:02 PAT: Off Voxel size: 1.5×1.5×1.5 mm Rel. SNR: 1.00 USER: cmrr_mbep2d_se

Properties

Prio Recon	Off
Before measurement	
After measurement	
Load to viewer	On
Inline movie	Off
Auto store images	On
Load to stamp segments	Off
Load images to graphic segments	Off
Auto open inline display	Off
Start measurement without further preparation	On
Wait for user to start	Off
Start measurements	single

Routine

Slice group 1	
Slices	90
Dist. factor	0 %
Position	Isocenter
Orientation	Transversal
Phase enc. dir.	A >> P
Rotation	0.00 deg
Phase oversampling	0 %
FoV read	192 mm
FoV phase	100.0 %
Slice thickness	1.50 mm
TR	15530 ms
TE	97.6 ms
Multi-band accel. factor	1
Filter	Raw filter
Coil elements	HEA;HEP

Contrast

MTC	Off
Magn. preparation	None
Flip angle	90 deg
Refocus flip angle	180 deg
Fat suppr.	Fat sat.
Grad. rev. fat suppr.	Enabled
Averaging mode	Long term
Reconstruction	Magnitude
Measurements	3
Delay in TR	0 ms
Multiple series	Off

Resolution

Base resolution	128
Phase resolution	100 %
Phase partial Fourier	7/8
Interpolation	Off
PAT mode	None
Matrix Coil Mode	Auto (CP)
Distortion Corr.	Off
Prescan Normalize	Off
Raw filter	On
Intensity	Weak
Slope	25
Elliptical filter	Off
Hamming	Off

Geometry

Multi-slice mode	Interleaved
Series	Interleaved
Special sat.	None

System

Body	Off
HEP	On
HEA	On
Positioning mode	REF
Table position	H
Table position	0 mm
MSMA	S - C - T
Sagittal	R >> L
Coronal	A >> P
Transversal	F >> H
Coil Combine Mode	Sum of Squares
AutoAlign	---
Auto Coil Select	Default
Shim mode	Standard
Adjust with body coil	Off
Confirm freq. adjustment	Off
Assume Silicone	Off
? Ref. amplitude 1H	0.000 V
Adjustment Tolerance	Auto
Adjust volume	
Position	Isocenter
Orientation	Transversal
Rotation	0.00 deg
R >> L	192 mm
A >> P	192 mm
F >> H	135 mm

Physio

1st Signal/Mode	None

BOLD

GLM Statistics	Off
Dynamic t-maps	Off
Starting ignore meas	0
Ignore after transition	0
Model transition states	On
Temp. highpass filter	On
Threshold	4.00
Paradigm size	15
Meas[1]	Baseline
Meas[2]	Baseline
Meas[3]	Baseline
Meas[4]	Baseline
Meas[5]	Baseline
Meas[6]	Baseline
Meas[7]	Baseline
Meas[8]	Baseline
Meas[9]	Baseline
Meas[10]	Baseline
Meas[11]	Active
Meas[12]	Active
Meas[13]	Active
Meas[14]	Active
Meas[15]	Active
Motion correction	Off
Spatial filter	Off

Sequence

Introduction	Off
Contrasts	1

Bandwidth	1776 Hz/Px
Free echo spacing	Off
Echo spacing	0.88 ms
EPI factor	128
RF pulse type	Normal
Gradient mode	Fast
Fake MB factor for SB	6
SENSE1 coil combine	Off
Invert RO/PE polarity	On
FFT scale factor	1.00
Physio recording	Legacy
Triggering scheme	Standard

\\USER\Dr Poppenk\fmri\MEMRI\T1w HCP

TA: 7:30 PAT: 2 Voxel size: 0.7×0.7×0.7 mm Rel. SNR: 1.00 SIEMENS: tfl

Properties

Prio Recon	Off
Before measurement	
After measurement	
Load to viewer	On
Inline movie	Off
Auto store images	On
Load to stamp segments	On
Load images to graphic segments	Off
Auto open inline display	Off
Start measurement without further preparation	On
Wait for user to start	Off
Start measurements	single

Routine

Slab group 1	
Slabs	1
Dist. factor	50 %
Position	R4.0 A39.3 F18.7
Orientation	Sagittal
Phase enc. dir.	A >> P
Rotation	0.00 deg
Phase oversampling	10 %
Slice oversampling	0.0 %
Slices per slab	256
FoV read	224 mm
FoV phase	100.0 %
Slice thickness	0.70 mm
TR	2400 ms
TE	2.13 ms
Averages	1
Concatenations	1
Filter	Prescan Normalize
Coil elements	HEA;HEP

Contrast

Magn. preparation	Non-sel. IR
TI	1000 ms
Flip angle	8 deg
Fat suppr.	None
Water suppr.	None
Averaging mode	Long term
Reconstruction	Magnitude
Measurements	1
Multiple series	Each measurement

Resolution

Base resolution	320
Phase resolution	100 %
Slice resolution	100 %
Phase partial Fourier	Off
Slice partial Fourier	Off
Interpolation	Off
PAT mode	GRAPPA
Accel. factor PE	2
Ref. lines PE	24
Accel. factor 3D	1
Matrix Coil Mode	Auto (Triple)
Reference scan mode	Integrated
Image Filter	Off
Distortion Corr.	Off
Unfiltered images	On
Prescan Normalize	On
Normalize	Off
B1 filter	Off
Raw filter	Off
Elliptical filter	Off

Geometry

Multi-slice mode	Single shot
Series	Ascending

System

Body	Off
HEP	On
HEA	On
Positioning mode	REF
Table position	H
Table position	0 mm
MSMA	S - C - T
Sagittal	R >> L
Coronal	A >> P
Transversal	F >> H
Save uncombined	Off
Coil Combine Mode	Adaptive Combine
AutoAlign	---
Auto Coil Select	Default
Shim mode	Tune up
Adjust with body coil	On
Confirm freq. adjustment	Off
Assume Silicone	Off
? Ref. amplitude 1H	0.000 V
Adjustment Tolerance	Auto
Adjust volume	
Position	Isocenter
Orientation	Transversal
Rotation	0.00 deg
R >> L	350 mm
A >> P	263 mm
F >> H	350 mm

Physio

1st Signal/Mode	None
Dark blood	Off
Resp. control	Off

Inline

Subtract	Off
Std-Dev-Sag	Off
Std-Dev-Cor	Off
Std-Dev-Tra	Off
Std-Dev-Time	Off
MIP-Sag	Off
MIP-Cor	Off
MIP-Tra	Off
MIP-Time	Off
Save original images	On

Sequence

Introduction	On
Dimension	3D
Elliptical scanning	Off
Asymmetric echo	Allowed
Bandwidth	240 Hz/Px
Flow comp.	No

Echo spacing	6.5 ms
RF pulse type	Fast
Gradient mode	Fast*
Excitation	Non-sel.
RF spoiling	On

\\USER\Dr Poppenk\fmri\MEMRI\T2 SPACE_3D

TA: 6:51 PAT: 2 Voxel size: 0.7×0.7×0.7 mm Rel. SNR: 1.00 SIEMENS: tse_vfl

Properties

Prio Recon	Off
Before measurement	
After measurement	
Load to viewer	On
Inline movie	Off
Auto store images	On
Load to stamp segments	Off
Load images to graphic segments	Off
Auto open inline display	Off
Start measurement without further preparation	On
Wait for user to start	Off
Start measurements	single

Routine

Slab group 1	
Slabs	1
Position	L14.9 P36.6 F20.3
Orientation	Sagittal
Phase enc. dir.	A >> P
Rotation	0.00 deg
Phase oversampling	10 %
Slice oversampling	0.0 %
Slices per slab	256
FoV read	224 mm
FoV phase	100.0 %
Slice thickness	0.70 mm
TR	3200 ms
TE	567 ms
Averages	1.0
Concatenations	1
Filter	Prescan Normalize
Coil elements	HEA;HEP

Contrast

MTC	Off
Magn. preparation	None
Fat suppr.	None
Water suppr.	None
Restore magn.	Off
Reconstruction	Magnitude
Measurements	1
Multiple series	Each measurement

Resolution

Base resolution	320
Phase resolution	100 %
Slice resolution	100 %
Phase partial Fourier	Allowed
Slice partial Fourier	Off
Interpolation	Off
PAT mode	GRAPPA
Accel. factor PE	2
Ref. lines PE	24
Accel. factor 3D	1
Matrix Coil Mode	Auto (Triple)
Reference scan mode	Integrated
Image Filter	Off
Distortion Corr.	Off
Unfiltered images	On
Prescan Normalize	On
Normalize	Off
B1 filter	Off
Raw filter	Off
Elliptical filter	Off

Geometry

Special sat.	None

System

Body	Off
HEP	On
HEA	On
Positioning mode	REF
Table position	H
Table position	0 mm
MSMA	S - C - T
Sagittal	R >> L
Coronal	A >> P
Transversal	F >> H
Save uncombined	Off
Coil Combine Mode	Adaptive Combine
AutoAlign	---
Auto Coil Select	Default
Shim mode	Standard
Adjust with body coil	Off
Confirm freq. adjustment	Off
Assume Silicone	Off
? Ref. amplitude 1H	0.000 V
Adjustment Tolerance	Auto
Adjust volume	
Position	L14.9 P36.6 F20.3
Orientation	Sagittal
Rotation	0.00 deg
F >> H	224 mm
A >> P	224 mm
R >> L	180 mm

Physio

1st Signal/Mode	None
Dark blood	Off
Resp. control	Off

Inline

Subtract	Off
Std-Dev-Sag	Off
Std-Dev-Cor	Off
Std-Dev-Tra	Off
Std-Dev-Time	Off
MIP-Sag	Off
MIP-Cor	Off
MIP-Tra	Off
MIP-Time	Off
Save original images	On

Sequence

Introduction	On
Dimension	3D
Bandwidth	744 Hz/Px
Flow comp.	No
Allowed delay	0 s
Echo spacing	3.74 ms
Adiabatic-mode	Off

Define	Echo trains
Turbo factor	189
Slice turbo factor	2
Echo trains per slice	1
Echo train duration	1268
RF pulse type	Normal
Gradient mode	Fast
Excitation	Non-sel.
Flip angle mode	T2 var

\\USER\Dr Poppenk\fmri\MEMRI\BOLD_PA_1.5iso-hippar

TA: 5:06 PAT: Off Voxel size: 1.5×1.5×1.5 mm Rel. SNR: 1.00 USER: cmrr_mbep2d_bold

Properties

Prio Recon	Off
Before measurement	
After measurement	
Load to viewer	On
Inline movie	Off
Auto store images	On
Load to stamp segments	Off
Load images to graphic segments	Off
Auto open inline display	Off
Start measurement without further preparation	On
Wait for user to start	Off
Start measurements	single

Routine

Slice group 1	
Slices	90
Dist. factor	0 %
Position	Isocenter
Orientation	Transversal
Phase enc. dir.	A >> P
Rotation	0.00 deg
Phase oversampling	0 %
FoV read	192 mm
FoV phase	100.0 %
Slice thickness	1.50 mm
TR	1900 ms
TE	36.8 ms
Multi-band accel. factor	6
Filter	Raw filter
Coil elements	HEA;HEP

Contrast

MTC	Off
Magn. preparation	None
Flip angle	75 deg
Fat suppr.	Fat sat.
Averaging mode	Long term
Reconstruction	Magnitude
Measurements	153
Delay in TR	0 ms
Multiple series	Off

Resolution

Base resolution	128
Phase resolution	100 %
Phase partial Fourier	6/8
Interpolation	Off
PAT mode	None
Matrix Coil Mode	Auto (CP)
Distortion Corr.	Off
Prescan Normalize	Off
Raw filter	On
Intensity	Weak
Slope	25
Elliptical filter	Off
Hamming	Off

Geometry

Multi-slice mode	Interleaved
Series	Interleaved
Special sat.	None

System

Body	Off
HEP	On
HEA	On
Positioning mode	REF
Table position	H
Table position	0 mm
MSMA	S - C - T
Sagittal	R >> L
Coronal	A >> P
Transversal	F >> H
Coil Combine Mode	Sum of Squares
AutoAlign	---
Auto Coil Select	Default
Shim mode	Standard
Adjust with body coil	Off
Confirm freq. adjustment	Off
Assume Silicone	Off
? Ref. amplitude 1H	0.000 V
Adjustment Tolerance	Auto
Adjust volume	
Position	Isocenter
Orientation	Transversal
Rotation	0.00 deg
R >> L	192 mm
A >> P	192 mm
F >> H	135 mm

Physio

1st Signal/Mode	None

BOLD

GLM Statistics	Off
Dynamic t-maps	Off
Starting ignore meas	0
Ignore after transition	0
Model transition states	On
Temp. highpass filter	On
Threshold	4.00
Paradigm size	20
Meas[1]	Baseline
Meas[2]	Baseline
Meas[3]	Baseline
Meas[4]	Baseline
Meas[5]	Baseline
Meas[6]	Baseline
Meas[7]	Baseline
Meas[8]	Baseline
Meas[9]	Baseline
Meas[10]	Baseline
Meas[11]	Active
Meas[12]	Active
Meas[13]	Active
Meas[14]	Active
Meas[15]	Active
Meas[16]	Active
Meas[17]	Active
Meas[18]	Active
Meas[19]	Active
Meas[20]	Active
Motion correction	Off
Spatial filter	Off

Sequence

Introduction	Off
Contrasts	1
Bandwidth	1776 Hz/Px
Flow comp.	No
Free echo spacing	Off
Echo spacing	0.88 ms
EPI factor	128
Gradient mode	Fast
RF spoiling	Off
Excite pulse duration	6300 us
Single-band images	On
MB LeakBlock kernel	Off
MB dual kernel	Off
MB RF phase scramble	Off
SENSE1 coil combine	Off
Invert RO/PE polarity	On
PF omits higher k-space	Off
Online multi-band recon.	Online
FFT scale factor	1.00
Physio recording	Legacy
Triggering scheme	Standard

\\USER\Dr Poppenk\fmri\MEMRI\DTI_RL_1.5iso

TA: 5:57 PAT: Off Voxel size: 1.5×1.5×1.5 mm Rel. SNR: 1.00 USER: cmrr_mbep2d_diff

Properties

Prio Recon	Off
Before measurement	
After measurement	
Load to viewer	On
Inline movie	Off
Auto store images	On
Load to stamp segments	Off
Load images to graphic segments	Off
Auto open inline display	Off
Start measurement without further preparation	On
Wait for user to start	Off
Start measurements	single

Routine

Slice group 1	
Slices	93
Dist. factor	0 %
Position	Isocenter
Orientation	Transversal
Phase enc. dir.	R >> L
Rotation	90.00 deg
Phase oversampling	0 %
FoV read	192 mm
FoV phase	100.0 %
Slice thickness	1.50 mm
TR	5180 ms
TE	103.4 ms
Multi-band accel. factor	3
Filter	Raw filter
Coil elements	HEA;HEP

Contrast

MTC	Off
Magn. preparation	None
Flip angle	78 deg
Refocus flip angle	160 deg
Fat suppr.	Fat sat.
Grad. rev. fat suppr.	Enabled
Averaging mode	Long term
Reconstruction	Magnitude
Measurements	1
Delay in TR	0 ms
Multiple series	Off

Resolution

Base resolution	128
Phase resolution	100 %
Phase partial Fourier	6/8
Interpolation	Off
PAT mode	None
Matrix Coil Mode	Auto (CP)
Distortion Corr.	Off
Prescan Normalize	Off
Raw filter	On
Intensity	Weak
Slope	25
Elliptical filter	Off
Hamming	Off

Geometry

Multi-slice mode	Interleaved
Series	Interleaved
Special sat.	None

System

Body	Off
HEP	On
HEA	On
Positioning mode	REF
Table position	H
Table position	0 mm
MSMA	S - C - T
Sagittal	R >> L
Coronal	A >> P
Transversal	F >> H
Coil Combine Mode	Sum of Squares
AutoAlign	---
Auto Coil Select	Default
Shim mode	Standard
Adjust with body coil	Off
Confirm freq. adjustment	Off
Assume Silicone	Off
? Ref. amplitude 1H	0.000 V
Adjustment Tolerance	Auto
Adjust volume	
Position	Isocenter
Orientation	Transversal
Rotation	90.00 deg
A >> P	192 mm
R >> L	192 mm
F >> H	140 mm

Physio

1st Signal/Mode	None

Diff

Diffusion mode	MDDW
Diff. weightings	2
b-value 1	0 s/mm²
b-value 2	1200 s/mm²
Diff. weighted images	On
Trace weighted images	On
Average ADC maps	On
Individual ADC maps	Off
FA maps	Off
Mosaic	Off
Tensor	Off
Noise level	40
Diff. directions	64

Sequence

Introduction	Off
Bandwidth	1502 Hz/Px
Free echo spacing	Off
Echo spacing	0.77 ms
EPI factor	128
Gradient mode	Fast
RF spoiling	Off
Excite pulse duration	2560 us
Refocus pulse duration	5760 us
Diffusion Scheme	Monopolar
Single-band images	On
MB LeakBlock kernel	Off
MB dual kernel	Off

MB RF phase scramble	Off
Time-shifted MB RF	Off
SENSE1 coil combine	On
Invert RO/PE polarity	Off
Online multi-band recon.	Online
FFT scale factor	1.00
Physio recording	Legacy

\\USER\Dr Poppenk\fmri\MEMRI\DTI_RL_1.5iso_b0

TA: 0:26 PAT: Off Voxel size: 1.5×1.5×1.5 mm Rel. SNR: 1.00 USER: cmrr_mbep2d_diff

Properties

Prio Recon	Off
Before measurement	
After measurement	
Load to viewer	On
Inline movie	Off
Auto store images	On
Load to stamp segments	Off
Load images to graphic segments	Off
Auto open inline display	Off
Start measurement without further preparation	On
Wait for user to start	Off
Start measurements	single

Routine

Slice group 1	
Slices	93
Dist. factor	0 %
Position	Isocenter
Orientation	Transversal
Phase enc. dir.	R >> L
Rotation	90.00 deg
Phase oversampling	0 %
FoV read	192 mm
FoV phase	100.0 %
Slice thickness	1.50 mm
TR	5180 ms
TE	103.4 ms
Multi-band accel. factor	3
Filter	Raw filter
Coil elements	HEA;HEP

Contrast

MTC	Off
Magn. preparation	None
Flip angle	78 deg
Refocus flip angle	160 deg
Fat suppr.	Fat sat.
Grad. rev. fat suppr.	Enabled
Averaging mode	Long term
Reconstruction	Magnitude
Measurements	1
Delay in TR	0 ms
Multiple series	Off

Resolution

Base resolution	128
Phase resolution	100 %
Phase partial Fourier	6/8
Interpolation	Off
PAT mode	None
Matrix Coil Mode	Auto (CP)
Distortion Corr.	Off
Prescan Normalize	Off
Raw filter	On
Intensity	Weak
Slope	25
Elliptical filter	Off
Hamming	Off

Geometry

Multi-slice mode	Interleaved
Series	Interleaved
Special sat.	None

System

Body	Off
HEP	On
HEA	On
Positioning mode	REF
Table position	H
Table position	0 mm
MSMA	S - C - T
Sagittal	R >> L
Coronal	A >> P
Transversal	F >> H
Coil Combine Mode	Sum of Squares
AutoAlign	---
Auto Coil Select	Default
Shim mode	Standard
Adjust with body coil	Off
Confirm freq. adjustment	Off
Assume Silicone	Off
? Ref. amplitude 1H	0.000 V
Adjustment Tolerance	Auto
Adjust volume	
Position	Isocenter
Orientation	Transversal
Rotation	90.00 deg
A >> P	192 mm
R >> L	192 mm
F >> H	140 mm

Physio

1st Signal/Mode	None

Diff

Diffusion mode	MDDW
Diff. weightings	1
b-value	0 s/mm²
Diff. weighted images	On
Trace weighted images	Off
Average ADC maps	Off
Individual ADC maps	Off
FA maps	Off
Mosaic	Off
Tensor	Off
Noise level	40
Diff. directions	64

Sequence

Introduction	Off
Bandwidth	1502 Hz/Px
Free echo spacing	Off
Echo spacing	0.77 ms
EPI factor	128
Gradient mode	Fast
RF spoiling	Off
Excite pulse duration	2560 us
Refocus pulse duration	5760 us
Diffusion Scheme	Monopolar
Single-band images	On
MB LeakBlock kernel	Off
MB dual kernel	Off
MB RF phase scramble	Off

Time-shifted MB RF	Off
SENSE1 coil combine	On
Invert RO/PE polarity	Off
Online multi-band recon.	Online
FFT scale factor	1.00
Physio recording	Legacy

\\USER\Dr Poppenk\fmri\MEMRI\DTI_LR_1.5iso

TA: 5:57 PAT: Off Voxel size: 1.5×1.5×1.5 mm Rel. SNR: 1.00 USER: cmrr_mbep2d_diff

Properties

Prio Recon	Off
Before measurement	
After measurement	
Load to viewer	On
Inline movie	Off
Auto store images	On
Load to stamp segments	Off
Load images to graphic segments	Off
Auto open inline display	Off
Start measurement without further preparation	On
Wait for user to start	Off
Start measurements	single

Routine

Slice group 1	
Slices	93
Dist. factor	0 %
Position	Isocenter
Orientation	Transversal
Phase enc. dir.	R >> L
Rotation	90.00 deg
Phase oversampling	0 %
FoV read	192 mm
FoV phase	100.0 %
Slice thickness	1.50 mm
TR	5180 ms
TE	103.4 ms
Multi-band accel. factor	3
Filter	Raw filter
Coil elements	HEA;HEP

Contrast

MTC	Off
Magn. preparation	None
Flip angle	78 deg
Refocus flip angle	160 deg
Fat suppr.	Fat sat.
Grad. rev. fat suppr.	Enabled
Averaging mode	Long term
Reconstruction	Magnitude
Measurements	1
Delay in TR	0 ms
Multiple series	Off

Resolution

Base resolution	128
Phase resolution	100 %
Phase partial Fourier	6/8
Interpolation	Off
PAT mode	None
Matrix Coil Mode	Auto (CP)
Distortion Corr.	Off
Prescan Normalize	Off
Raw filter	On
Intensity	Weak
Slope	25
Elliptical filter	Off
Hamming	Off

Geometry

Multi-slice mode	Interleaved
Series	Interleaved
Special sat.	None

System

Body	Off
HEP	On
HEA	On
Positioning mode	REF
Table position	H
Table position	0 mm
MSMA	S - C - T
Sagittal	R >> L
Coronal	A >> P
Transversal	F >> H
Coil Combine Mode	Sum of Squares
AutoAlign	---
Auto Coil Select	Default
Shim mode	Standard
Adjust with body coil	Off
Confirm freq. adjustment	Off
Assume Silicone	Off
? Ref. amplitude 1H	0.000 V
Adjustment Tolerance	Auto
Adjust volume	
Position	Isocenter
Orientation	Transversal
Rotation	90.00 deg
A >> P	192 mm
R >> L	192 mm
F >> H	140 mm

Physio

1st Signal/Mode	None

Diff

Diffusion mode	MDDW
Diff. weightings	2
b-value 1	0 s/mm²
b-value 2	1200 s/mm²
Diff. weighted images	On
Trace weighted images	On
Average ADC maps	On
Individual ADC maps	Off
FA maps	Off
Mosaic	Off
Tensor	Off
Noise level	40
Diff. directions	64

Sequence

Introduction	Off
Bandwidth	1502 Hz/Px
Free echo spacing	Off
Echo spacing	0.77 ms
EPI factor	128
Gradient mode	Fast
RF spoiling	Off
Excite pulse duration	2560 us
Refocus pulse duration	5760 us
Diffusion Scheme	Monopolar
Single-band images	On
MB LeakBlock kernel	Off
MB dual kernel	Off

MB RF phase scramble	Off
Time-shifted MB RF	Off
SENSE1 coil combine	On
Invert RO/PE polarity	On
Online multi-band recon.	Online
FFT scale factor	1.00
Physio recording	Legacy

\\USER\Dr Poppenk\fmri\MEMRI\DTI_LR_1.5iso_b0

TA: 0:26 PAT: Off Voxel size: 1.5×1.5×1.5 mm Rel. SNR: 1.00 USER: cmrr_mbep2d_diff

Properties

Prio Recon	Off
Before measurement	
After measurement	
Load to viewer	On
Inline movie	Off
Auto store images	On
Load to stamp segments	Off
Load images to graphic segments	Off
Auto open inline display	Off
Start measurement without further preparation	On
Wait for user to start	Off
Start measurements	single

Routine

Slice group 1	
Slices	93
Dist. factor	0 %
Position	Isocenter
Orientation	Transversal
Phase enc. dir.	R >> L
Rotation	90.00 deg
Phase oversampling	0 %
FoV read	192 mm
FoV phase	100.0 %
Slice thickness	1.50 mm
TR	5180 ms
TE	103.4 ms
Multi-band accel. factor	3
Filter	Raw filter
Coil elements	HEA;HEP

Contrast

MTC	Off
Magn. preparation	None
Flip angle	78 deg
Refocus flip angle	160 deg
Fat suppr.	Fat sat.
Grad. rev. fat suppr.	Enabled
Averaging mode	Long term
Reconstruction	Magnitude
Measurements	1
Delay in TR	0 ms
Multiple series	Off

Resolution

Base resolution	128
Phase resolution	100 %
Phase partial Fourier	6/8
Interpolation	Off
PAT mode	None
Matrix Coil Mode	Auto (CP)
Distortion Corr.	Off
Prescan Normalize	Off
Raw filter	On
Intensity	Weak
Slope	25
Elliptical filter	Off
Hamming	Off

Geometry

Multi-slice mode	Interleaved
Series	Interleaved
Special sat.	None

System

Body	Off
HEP	On
HEA	On
Positioning mode	REF
Table position	H
Table position	0 mm
MSMA	S - C - T
Sagittal	R >> L
Coronal	A >> P
Transversal	F >> H
Coil Combine Mode	Sum of Squares
AutoAlign	---
Auto Coil Select	Default
Shim mode	Standard
Adjust with body coil	Off
Confirm freq. adjustment	Off
Assume Silicone	Off
? Ref. amplitude 1H	0.000 V
Adjustment Tolerance	Auto
Adjust volume	
Position	Isocenter
Orientation	Transversal
Rotation	90.00 deg
A >> P	192 mm
R >> L	192 mm
F >> H	140 mm

Physio

1st Signal/Mode	None

Diff

Diffusion mode	MDDW
Diff. weightings	1
b-value	0 s/mm²
Diff. weighted images	On
Trace weighted images	Off
Average ADC maps	Off
Individual ADC maps	Off
FA maps	Off
Mosaic	Off
Tensor	Off
Noise level	40
Diff. directions	64

Sequence

Introduction	Off
Bandwidth	1502 Hz/Px
Free echo spacing	Off
Echo spacing	0.77 ms
EPI factor	128
Gradient mode	Fast
RF spoiling	Off
Excite pulse duration	2560 us
Refocus pulse duration	5760 us
Diffusion Scheme	Monopolar
Single-band images	On
MB LeakBlock kernel	Off
MB dual kernel	Off
MB RF phase scramble	Off

Time-shifted MB RF	Off
SENSE1 coil combine	On
Invert RO/PE polarity	On
Online multi-band recon.	Online
FFT scale factor	1.00
Physio recording	Legacy
